# Recent Advancements in Electroporation Technologies: From Bench to Clinic

**DOI:** 10.1146/annurev-bioeng-110220-023800

**Published:** 2023-02-28

**Authors:** Sabrina N. Campelo, Po-Hsun Huang, Cullen R. Buie, Rafael V. Davalos

**Affiliations:** 1 Department of Biomedical Engineering and Mechanics, Virginia Tech–Wake Forest School of Biomedical Engineering and Sciences, Virginia Tech, Blacksburg, Virginia, USA;; 2 Department of Mechanical Engineering, Massachusetts Institute of Technology, Cambridge, Massachusetts, USA

**Keywords:** pulsed field ablation, transfection, gene therapy, electrochemotherapy, microfluidics

## Abstract

Over the past decade, the increased adoption of electroporation-based technologies has led to an expansion of clinical research initiatives. Electroporation has been utilized in molecular biology for mammalian and bacterial transfection; for food sanitation; and in therapeutic settings to increase drug uptake, for gene therapy, and to eliminate cancerous tissues. We begin this article by discussing the biophysics required for understanding the concepts behind the cell permeation phenomenon that is electroporation. We then review nano- and microscale single-cell electroporation technologies before scaling up to emerging in vivo applications.

## 1. INTRODUCTION

Electroporation is a biophysical phenomenon in which an external electric field generated around a cell increases cell permeability by disrupting the physical structure of the cell membrane. This physical principle has been applied in a wide range of applications, including in molecular biology for mammalian and bacterial gene transfection; in bacteria deactivation for food sanitation; and in clinical settings for increasing drug uptake and gene therapies, ablating undesirable tissues, and stimulating immune responses. The ability to put agents into cells while only temporarily disrupting the cell membrane is highly desirable and thus has rapidly developed in recent years. This application is termed reversible electroporation (RE) and may also be referred to as electropermeabilization or electrotransfection.

The first practical applications of electroporation came to light in the 1950s and 1960s after the discovery of its ability to inactivate bacteria, which was used extensively for food sterilization. In the 1980s, these efforts escalated to in vitro laboratory RE applications that enabled foreign substances such as DNA and altered genes to be inserted into cells. The early 1990s brought one of the most established applications of RE to date, electrochemotherapy (ECT), in which large, cytotoxic drug molecules are more readily taken up by tumor cells in vivo due to the increased permeability arising from the induced electric field. Prior reviews covering RE have focused on specific RE applications such as ECT or electrogene transfer (EGT) (1, 2). Others have highlighted the advancements of RE in a particular organ region or tumor morphology (3, 4). This review presents a broad overview of RE applications, starting at the single-cell level in an in vitro setup and scaling up to full-size multicellular in vivo organ studies. We present a brief overview of the relevant mathematics and biophysics, followed by a general description of different RE modalities in vitro and in vivo. While our discussion focuses on RE modalities and their applications, we highlight how they compare with competing techniques for the same application.

## 2. BIOPHYSICAL THEORY OF ELECTROPORATION

Understanding the theory behind electroporation is critical for the development and optimization of biomedical applications utilizing this phenomenon. Electroporation is the process in which applied external electric fields stimulate an increase in cellular membrane permeability and pore formation (5, 6). This biophysical induction comes as the result of applied fields triggering an increase in the cellular transmembrane potential (TMP). The typical resting potential of a healthy eukaryotic cell is approximately −0.07 V due to a naturally occurring osmotic gradient. However, if the TMP exceeds a particular critical transmembrane threshold (TMP*), typically between 0.2 and 0.5 V, then ions and other macromolecules can permeate the cellular membrane as a result of structural defects within the phospholipid bilayer. This threshold is dependent on characteristics of the applied pulses and tissue properties.

### 2.1. Cellular Electroporation

Before advancing into the biophysics defining electropermeabilization, one must first understand a simplified model of the cell. The cell comprises an inner-conducting cytoplasm enveloped by a surrounding conducting medium. These two components are separated by an electrically insulating phospholipid bilayer, consisting of hydrophilic heads and hydrophobic tails, responsible for the physical act of thermodynamically driven pore formation.

**Figure 1:**
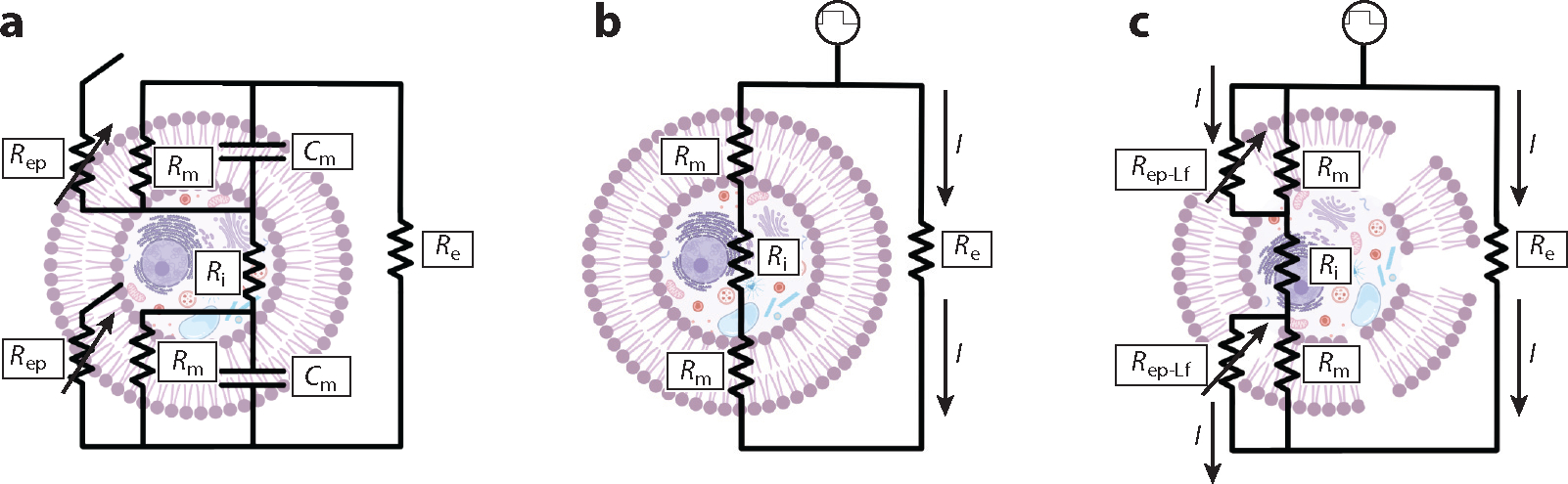
Equivalent cell models may be used to represent the electrical pathways of the intact cell (*a*) prior to pulsing, (*b*) while exposed to a low-frequency unipolar pulse prior to membrane disruption, and (*c*) following electroporation leading to membrane impedance reduction with low-frequency unipolar bursts. In all cases, current travels down the paths of least resistance. Abbreviations: $C_{m}$, capacitance of the cellular membrane; I, current; $R_{e}$, extracellular resistance; $R_{ep}$, resistance of the membrane undergoing electroporation; $R_{ep\text{-Lf}\;}$, reduced resistance of electroporated membrane under low-frequency current; $R_{i}$, intracellular resistance; $R_{m}$, resistance of the intact cell membrane. Figure adapted from images created with BioRender.com.

A cell can be modeled through a simplified circuit model consisting of a series of resistors representing the extracellular resistance $\left(R_{e}\right)$, the intracellular resistance $\left(R_{i}\right)$, and the capacitance of the cellular membrane $\left(C_{m}\right)$ (Figure 1a). However, the cellular membrane is not fully dielectric and becomes partially conductive under the influence of electroporation. Figure 1b depicts the equivalent circuit for a cell undergoing bursts of low-frequency unipolar pulses [8 pulses for ECT (7, 8) and 80–100 for irreversible electroporation (IRE) (9)]. As pores begin to form, current can travel through a wider variety of transmembrane pathways, thereby reducing the overall effective resistance $\left(R_{ep}∥R_{m}\right)$, where $R_{ep}$ is the variable resistance of the membrane undergoing electroporation in parallel with the resistance of the intact cell membrane ($R_{m}$) (Figure 1c). At high enough frequencies, the circuit is shorted for the current to travel directly through the intracellular and extracellular spaces, whereas lower-frequency pulses cause the current to travel predominantly around the extracellular perimeters until pore formation becomes dominant (10, 11).

Once the TMP* voltage is exceeded, membrane breakdown occurs in two phases (12):

1. Extracellular medium enters through transient hydrophobic pores formed as a result of dielectric cellular membrane breakdown and thermal fluctuations.
2. If the pulsing parameters result in a high TMP exceeding a critical threshold (~0.5 V), it becomes more energetically favorable for the hydrophilic heads to turn inward to face one another, creating reversible hydrophilic pores.
3. Should the TMP surpass an even higher threshold (~1 V), the cell will be unable to recover and will die as a result of irrecoverable damage.

The degree to which the TMP exceeds the threshold determines the extent of membrane disruption. If the pulse amplitude and duration are combined to permit pore resealing, and cell viability is still maintained after the pore closure, this process is referred to as RE. If the applied current is such that the cell cannot recover and dies, this process is referred to as IRE (12). Note that the membrane may revert to a standard closed bilayer state; however, the extended duration of time spent in the hydrophilic pore state leads to extended disruption of cell homeostasis triggering cell death. The abovementioned TMP is calculated using the Schwan equation (13):

1. $$TMP=f\cdot E\cdot r\cdot cos\varphi \cdot (1-e^{-\frac{t}{\tau }}),$$

where

2. $$f=\frac{3\sigma_{e}\left[3d_{m}r^{2}\sigma_{i}+\left(3d_{m}^{2}r-d_{m}^{3}\right)\left(\sigma_{m}-\sigma_{i}\right)\right]}{2r^{3}\left(\sigma_{m}+2\sigma_{e}\right)(\sigma_{m}+\frac{1}{2}\sigma_{i})-2{\left(r-d_{m}\right)}^{3}\left(\sigma_{e}-\sigma_{m}\right)\left(\sigma_{i}-\sigma_{m}\right)}$$

and

3. $$\tau =r\cdot C_{m}\left(\rho_{i}+\frac{\rho_{e}}{2}\right).$$

The conductivity of the extracellular medium, cell membrane, and cytoplasm is represented by $\sigma_{e},$ $\sigma_{m}$, and $\sigma_{i}$, respectively, and $d_{m}$ represents the cell membrane thickness. In the case of a single cell in suspension, f is a form factor describing the impact of the cell on the extracellular field distribution (e.g., 1.5 for a perfectly spherical cell), E is the applied electric field, r is the cell radius, and φ is the polar angle measured from the center of the assumed spherical cell in relation to the direction of the electric field. This relation demonstrates that geometric contributions from the cell shape and polar orientation play an integral role in manipulating the induced TMP. Given the relation in Equation 1, we can infer that a larger cell radius would require a lower applied electric field to induce hydrophilic pores (e.g., the electric field required to induce hydrophilic pores in mammalian cells is lower than in that of bacteria). Cell areas facing the electrodes deploy a markedly higher voltage drop across the intra- and extracellular membranes, making them more readily electroporated (14).

It is understood that $\sigma_{e}$ and $\sigma_{i}$ are held constant, while $\sigma_{m}$ changes as a function of the electric field. The following relation assumes that the change in membrane conductivity is due to the creation of pores in the membrane (15):

4. $$\sigma_{m}\left(V_{m}\right)=\sigma_{m0}+\sigma_{\text{pores}\;}\left[\lambda \left(e^{\beta \left|V_{m}\right|}-1\right)\right],$$

where $\sigma_{m0}$ is the initial conductivity of the membrane when the applied voltage $V_{m}$ is zero; $\sigma_{\text{pores}}$ is the conductivity of the medium that fills the pores, estimated as the average value of $\sigma_{e}$ and $\sigma_{i}$; and both λ and β are constants describing how the membrane conductivity increases along with the TMP. The last term in the function, $\sigma_{\text{pores}}\left[\lambda \left(e^{\beta \left|V_{m}\right|}-1\right)\right]$, represents the relative area of the pores.

In addition to the spatial component of the cell, the cellular membrane is often modeled as a capacitor, suggesting that a charging delay exists between the time an electric field is applied and when a change in transmembrane voltage is induced. In Equation 3, τ is the charging time constant of the cell membrane on the order of 1 μs or less, $C_{m}$ is the capacitance of the cell membrane per unit area, and $\rho_{i}$ and $\rho_{e}$ are the resistivities of the intracellular and extracellular domains, respectively (16). The time-dependent Schwan equation demonstrates contributions from the time-based capacitive membrane. Due to the charging time, a short pulse on the order of τ will not cause electroporation of the cellular membrane unless the applied electric field is extremely high. High-magnitude submicrosecond pulses have demonstrated significant effects on cellular organelles such as the mitochondria (17).

Physically, while under an induced TMP*, the bilayer undergoes two stages, expansion and stabilization. While the transmembrane voltage remains above the critical value for a given cell (TMP > TMP*), the pore continues to expand, thus increasing permeability and conductivity. A smaller increase causes a reversible breakdown of the membrane, whereas a larger increase, typically on the order of 1 V, results in an irreparable breakdown that causes cell death (18). Stabilization occurs once the applied electric field is removed and the TMP drops down below the critical threshold (TMP < TMP*). The affected region of the cell membrane experiences a drop in conductivity, and the formed pores begin to reseal. Recovery occurs on the order of seconds to minutes at physiologic temperatures (19, 20). The sealing kinetics take time, as the cell must first displace the intervening medium out of the formed hydrophilic pore in order to allow the constituents of the lipid bilayer to return to their normal energy state.

### 2.2. Tissue Electroporation

The stochastic process of pore formation is influenced by various factors, such as electric field distribution, pulse parameters and shape, specific tissue properties, and temperature. Dynamic conductivity models are able to incorporate parametric factors that increase the conductivity of a medium during a series of pulses. The extracellular membrane conductance increases with each pulse applied, affecting the field distribution and increasing the electroporated area due to a reduction in the minimum applied electric field required to induce electroporation.

A range of factors contribute to the observed change in conductivity (21): (*a*) an increase in conductivity due to an increase in temperature (Equation 7), (*b*) an increase in conductivity due to an influx of ions leaking out of the intracellular matrix, (*c*) a transient increase in conductivity observed during the on-time of each pulse, and (*d*) a decrease in conductivity due to osmotic swelling of the cell. A solution to a modified Laplace equation that accounts for electroporation-induced conductivity changes resulting from the formation of nanopores is modeled by

5. ∇⋅[σ(E)∇ϕ]=0,

where ϕ is the electric potential allowing us to model the electric field distribution (−∇ϕ) within a tissue. Conductivity changes due to temperature increases may also be accounted for, as the temperature is affected by Joule heating. The associated change in temperature (ΔT) may be calculated as follows:

6. $$\Delta T=\frac{\sigma }{\rho c_{p}}|\nabla \phi {|}^{2}\Delta t,$$

where ρ is the tissue density, $c_{p}$ is the specific heat of the tissue, and Δt refers to the total duration of the pulses. This conservative estimate, which assumes no heat dissipation within the tissue, may be used to calculate the final conductivity σ(T) from the initial static conductivity $\sigma_{0}$ by the following equation:

7. $$\sigma \left(T\right)=\sigma_{0}\left[1+\alpha \cdot \Delta T\right],$$

where α is the temperature coefficient of the medium. The dynamic model considers the influences of electroporation to spread the electric field away from the electrodes, which has led to higher observed thresholds for inducing electroporation.

Despite many publications on the mechanisms of electroporation, a comprehensive understanding is still lacking. Complex models simulating the biophysical mechanisms of electroporation may utilize the abovementioned principles to simulate electric field distributions; however, the topics presented here do not encompass all of the variables that play a role in the mechanisms of electroporation. Determining which parameters are most significant in the context of the experimental or clinical objectives and incorporating them into the development of a comprehensive mathematical model are crucial. Understanding these phenomena is essential for the efficient use of electroporation in in vitro cell transfection, in vivo clinical transfection, and treatment planning. For additional information about the theory behind electroporation, we refer the reader to previous articles on the subject (20, 22, 23).

## 3. IN VITRO ELECTROPORATION

In the modern era of genomic sciences, transfection has become a critical tool for both basic research and clinical applications. Interest in transfection has been especially influenced by the development of nonviral gene editing modalities over the past 15–20 years, including transcription activator–like effector nucleases (24), zinc-finger nucleases (25, 26), transposons such as Sleeping Beauty (27), and more recently CRISPR-based editing approaches (28, 29). Transfection is an alternative to viral vector–mediated genetic manipulation that offers several advantages. First, production of viral vectors can be costly and time intensive, especially in the case of clinical-scale applications of gene editing where viral vector production has become a key bottleneck for scalability. Second, the payload size that can be delivered using viral vectors is limited to anywhere from 5 to 7 kb, depending on the viral vector of interest, limiting the functional change that can be induced in a single round of genetic manipulation. As researchers desire to impose more complex changes on cells and biological systems, this payload limitation becomes more pronounced. Lastly, some researchers within the field have lingering concerns that viral vector–based transduction may have long-term safety concerns for clinical gene therapy and adoptive cell therapy applications. As a result, there has been a recent surge in research and development of nonviral transfection methods, including a renewed interest in electroporation for both basic research and clinical applications.

### 3.1. Non-Electroporation-Based In Vitro Cell Transfection Methods

Relative to viral transduction, electroporation is relatively safe and inexpensive, particularly when one factors in the extensive development time required for viral vectors (30, 31). The relative simplicity of traditional cuvette/batch-based electroporation makes this approach attractive from a cost perspective. Furthermore, given the growing demand for nonviral transfection methods, it is useful to briefly discuss other transfection methodologies under development. We encourage the reader to consult the associated references for more information and details on these methods (32, 33).

Nonviral transfection methods can be broadly categorized into two themes: one in which the cell envelope is subjected to some form of energetic perturbation that leads to pore formation and another in which the cell envelope experiences a chemical or biochemical insult that compromises membrane integrity. Electroporation is a member of the first group, but there are other technologies that leverage different energetic inputs to induce pore formation. Several technologies can be classified as mechanical in nature, in that they impose a physical stress on the cell envelope to deliver exogenous materials. With the advent of nanofabrication, several research teams have shown that nanoscale needles, in an array of configurations, can be utilized to puncture the cell membrane for payload delivery; these are discussed in detail in Section 3.3.2, below. A second method involves the use of mechanical stress to induce pore formation in various types of cells in suspension (34–36). These technologies leverage the mechanical forces (e.g., shear stress and pressure) exerted on a cell as it flows in a fluid traveling at moderate Reynolds numbers (usually in the laminar flow regime). One of the major advantages of these systems is that, depending on their geometric configuration, the physics of this technology readily scales from small research volumes to larger clinically relevant volumes. A third mechanical method is known as cell squeezing (37). This technology is able to deliver an array of materials into cells while maintaining high cell viability and minimizing phenotypic perturbation (38). A fourth mechanical method of transfection that has been explored in the literature is the use of acoustic waves to permeabilize the cell membrane (39). This technology is still in its infancy from a development perspective, and whether the challenges associated with fabrication will be offset by future phenotypic or functional outcomes of cells transfected in this manner has yet to be determined.

The next class of nonviral transfection technologies utilizes chemical or biochemical compounds that perturb the cell membrane and lead to pore formation. Several technologies within this class of nonviral transfection platforms are in various stages of development. On the chemical side, studies have shown that solvents can be sprayed in a controlled way that leads to cell membrane permeabilization (40). This technology is being commercialized and has several useful features, including simplicity of design and relatively low cost requirements. With respect to biological compounds, studies have also shown that certain peptides (41) can be effective in permeabilizing cells for gene delivery. The key drawbacks for each of these technologies are potential challenges with delivery to the nucleus, as well as diffusion limitations related to the payload being delivered.

### 3.2. Electroporation-Mediated Gene Manipulation

Electroporation has become a key tool for both basic science and clinical research to leverage genetic manipulation. The flexibility, ease of use, and relative simplicity of electroporation-based transfection have made it one of the leading nonviral gene editing modalities. The following discussion provides a few examples of the use of electroporation for in vitro studies, with a scope ranging from fundamental knowledge to therapeutic development.

#### 3.2.1. Electroporation facilitates functional genomics.

A key advantage of in vitro electroporation for transfection is that the general methodology can be applied to an exceedingly broad range of cell types and payload combinations. Examples include primary human T cells (42), mesenchymal stem cells (43), THP-1 macrophages (44, 45), primary human monocytes (46), human embryonic stem cells (47, 48), human neural stem cells (49), and hematopoietic stem cells (50). Electroporation has experienced a technical resurgence that has been coupled with the explosion of interest in functional genomics. Nonviral transfection methods such as electroporation provide the scientific community with a relatively inexpensive research tool to investigate functional relationships between genes, cell function, and protein expression. Given the large number of cell types amenable to electroporation, it has become a valuable tool in both basic and applied research. For example, electroporation-based delivery of small interfering RNA can be used to selectively regulate gene expression through RNA interference (51) and can facilitate high-throughput genomic screening (52). These methods have been used in a wide variety of applications, ranging from understanding cell death mechanisms in bacterial infections (53) to immunosuppression in the tumor microenvironment (54). Homologous recombination can also be used to expose gene function relationships (55) and has been implemented using CRISPR/Cas9 ribonucleoprotein delivered by electroporation in combination with rAAV6 (recombinant adeno-associated virus serotype 6) in hematopoietic stem cells (50). Electroporation has proven particularly useful in cell types that are less amenable to viral transduction, including many primary cell types.

#### 3.2.2. Electroporation for genetic engineering and gene editing.

One of the most exciting therapeutic areas is that of adoptive cell therapy, in which human cells (either autologous or allogeneic) are delivered to a patient for treatment. The therapy can consist of unaltered cells from a healthy donor or genetically engineered cells. Electroporation can be utilized in both of these situations. Electroporation has been used to generate antigen-presenting cells from peripheral blood mononuclear cells (PBMCs) to study the engraftment of human central memory–derived effector CD8^+^ T cells for adoptive cell therapy (56). The transfected PBMCs were used to stimulate the T cells, leading to the finding that central memory T cells showed improved engraftment in comparison to effector memory T cells in a humanized mouse model. Early efforts in gene therapy utilized viral vectors but resulted in vector-related leukemia, a challenge that is mitigated with non-viral vector–based approaches that leverage electroporation (57, 58). When stable gene expression is required, early research in gene editing found that transposons such as Sleeping Beauty offer a viable delivery mechanism for DNA insertion (59). Since their initial discovery, there have been several advances in the development of transposons to optimize them for gene editing (27, 60). Early studies showed that Sleeping Beauty–based plasmid insertion can be used as an alternative to lentiviral vectors when engineering primary human T cells for antitumor activity (61). Unlike with viral vectors, transposition is not preferential to high-activity areas of the genome and is more random, which may reduce the potential for genotoxicity when compared with viral vectors. Given the potential safety benefits, Sleeping Beauty transposons in the form of minicircle DNA vectors have also been utilized to engineer CD19 chimeric antigen receptor (CAR) T cells (62). The minicircle format demonstrated stable genomic integration but with lower toxicity than plasmids, which had typically been used with transposons. These engineered CAR T cells exhibited anticancer cell activity both in vitro and in vivo.

While research with transposons continues, recent efforts have focused largely on delivering CRISPR constructs, particularly CRISPR/Cas9. Various CRISPR-based technologies have been used along with electroporation for both knockout and knock-in applications. Electroporation has been coupled with CRISPR/Cas9 genome targeting for insertion of DNA into primary human T cells (42). This research has demonstrated correction of a pathogenic mutation in cells from patients with a monogenic autoimmune disease and replacement of an endogenous T cell receptor (TCR) locus with an engineered TCR in T cells targeting a cancer antigen. Notably, the engineered TCR T cells killed the cancer cells in vitro. Electroporation has also been used as part of a screening methodology known as SLICE (single-guide RNA lentiviral infection with Cas9 protein electroporation) to perform functional studies in primary human T cells (63). Previously, CRISPR screens in primary human T cells had been challenging due to the low viral transduction efficiencies encountered with lentiviral vectors. This study coupled lentiviral single-guide RNA delivery with electroporation of Cas9 proteins to perform genome-scale loss-of-function screens. The results revealed that the SLICE methodology could identify targets that improved anticancer activity in vitro. Another study used a CRISPR pooled screening platform with primary mouse regulatory T cells (Tregs) to identify genetic modification associated with *Foxp3* regulation, which itself is a master regulator of Treg function (64). This study identified several positive and negative regulators of *Foxp3* that could serve as potential targets for Treg-based immunotherapies. Other studies have combined viral transduction with electroporation to conduct a CRISPR screen, using an in vitro assay, to identify key factors that lead to T cell exhaustion (65).

One of the most promising applications of electroporation and CRISPR-based gene editing has made its way to the clinic and is advancing toward approval. Here, electroporation and CRISPR/Cas9 are combined to edit the gene *BCL11A* and induce production of fetal hemoglobin in patients with sickle cell disease and β-thalassemia (66). Early clinical results are promising, with very favorable initial outcomes for the patients in the study.

### 3.3. Micro- and Nanoscale Electroporation

Over the past two decades, micro- and nanotechnologies (or micro- and nanofabrication) have emerged as alternative promising tools for a wide variety of chemical and biological applications. Examples include characterizing enzyme kinetics, recapitulating the tumor microenvironment for cancer metastasis, and profiling single-cell dynamics. Similarly, these tools have benefited the advancement of in vitro electroporation because of the ease of miniaturizing the size of electroporation electrodes and chambers and the capability of handling single or multiple cells at a time. The following subsections discuss how micro- and nanotechnologies have contributed to in vitro electroporation, ranging from high-throughput electroporation to targeted, single-cell electroporation.

#### 3.3.1. Microfluidic electroporation.

Due to their unique ability to achieve rapid and sensitive analysis with small sample and reagent volumes (67), microfluidic electroporation devices, in comparison to batchwise bulk electroporation (i.e., electroporation cuvettes), offer advantages that can address problems associated with cuvette-based techniques. First, miniaturized electrodes of microfluidic devices facilitate the use of significantly lower voltages to establish electric fields sufficient to transiently disrupt cell membranes. The miniaturized electrodes and reduced voltages can provide a uniform field while minimizing adverse effects such as bubble generation and Joule heating. Second, the surface area–to–volume ratio and reduced sample volume required in microfluidic devices lead to superior heat dissipation. Third, the use of transparent materials (e.g., polydimethylsiloxane, glass, quartz) enables the exploration and optimization of the electroporation conditions through in situ observation and real-time monitoring of the transfection process (68). Fourth, microfluidic approaches offer the design flexibility to alter microchannel dimensions and electrode configurations. One can readily adjust the position and dimension of electrodes or microchannels to establish a locally enhanced electric field so as to improve transfection efficiency or enable single-cell electroporation. Lastly, microfluidic devices can perform electroporation in a flow-through, continuous manner to assist in heat dissipation and improve overall throughput. These features can improve transfection efficiency and cell viability by overcoming certain issues associated with conventional electroporation, thus rendering microfluidic approaches promising for applications where high transfection rates and viability are required. A wide range of microfluidics-based electroporation devices have been discussed in a number of review articles (69, 70). This review focuses on devices that are intended for electroporation in a continuous manner and at the single-cell level.

**Figure 2:**
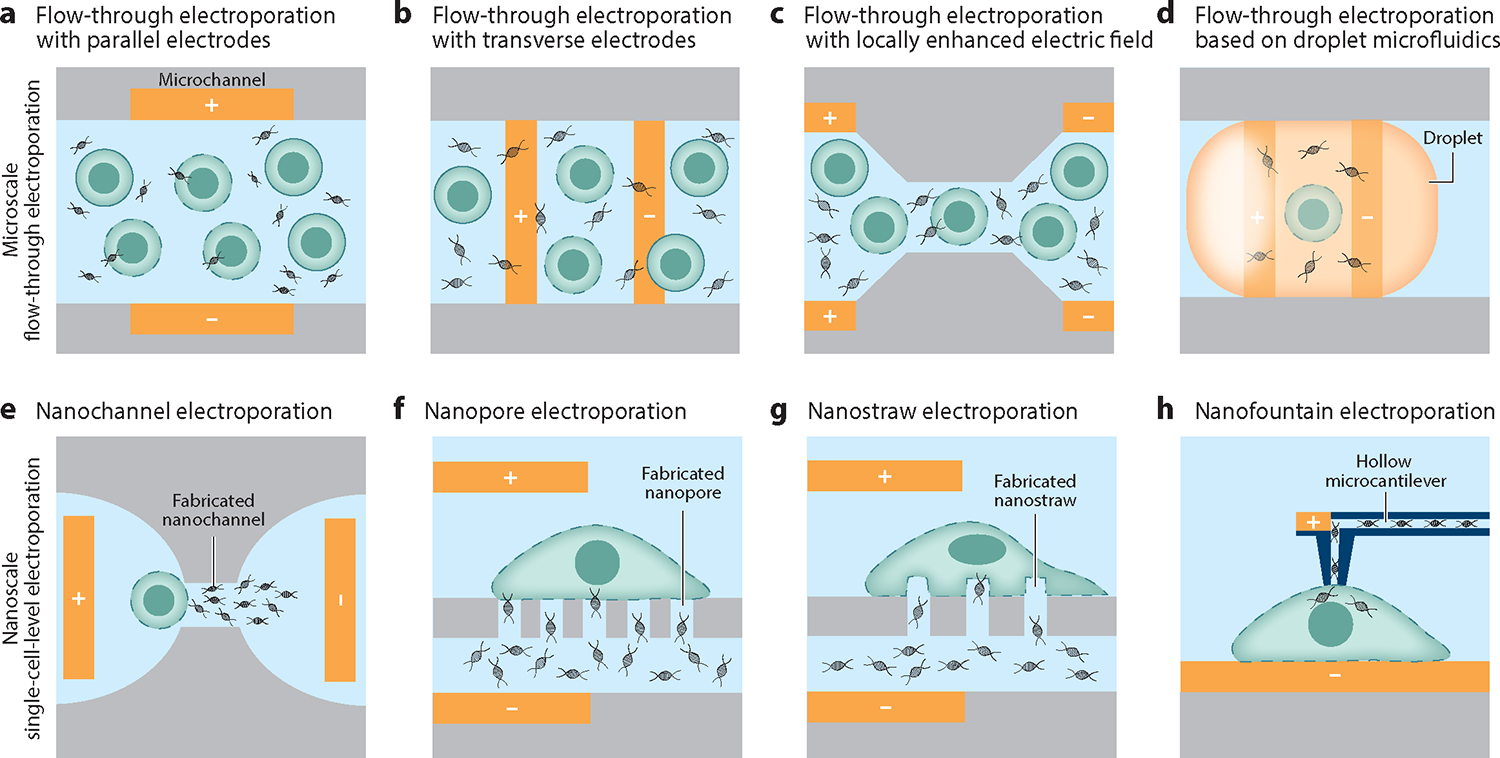
(*a*–*d*) Representative flow-through, microfluidics-based electroporation techniques and (*e*–*h*) nanostructure-mediated electroporation techniques at the single-cell level.

The concept of flow-through electroporation was first proposed in 2001 (71), in a study employing a microchip with two parallel gold-plate electrodes (Figure 2a). With this microchip, cells flowing through the region sandwiched by the two electrodes were electroporated in a continuous flow under a range of flow rates. Since then, numerous attempts have been made to locally enhance electric fields in microfluidic electroporation devices by modifying electrode design (Figure 2b) or microchannels (69, 70) in order to improve efficiency, throughput, and cell viability. Modifying the microchannel design is the most common way to locally enhance the field strength, where a major microchannel of a given width (wider channel) is designed with locally constricted microchannels to facilitate transfection (72, 73) (Figure 2c). The narrower microchannel can be close to the size of a single cell (10–20 μm), allowing the passage of multiple cells, and the width difference between the wider and narrower microchannels is large enough to generate locally enhanced electric fields. For example, Lu and colleagues (72, 73) reported a microfluidic electroporation device with five constricted regions in series so that cells could be subjected to five electrical pulses while applying only one continuous dc voltage source. Using this device, Chinese hamster ovary cells were transfected with pEGFP-C1 plasmid DNA with a transfection efficiency of ~75% at lower flow rates (<2 mL/min) and at higher flow rates up to ~20 mL/min, although at higher flow rates the transfection efficiency was compromised.

Similar concepts have been employed for reversible and irreversible bacterial electroporation (68, 74). Other efforts have been conducted to improve the scalability of flow-through, microfluidics-based electroporation platforms. For example, Tandon and colleagues (75) created an integrated device capable of transferring cells to be transfected from culture media to electroporation buffers and then immediately electroporating the cells, all in a continuous, flow-through manner. Using this integrated device, they demonstrated medium exchange for primary human T cells from a standard culture medium to an electroporation buffer, with a medium exchange efficiency of ~86%, and subsequently transfected T cells with messenger RNA–encoding fluorescent protein at efficiencies of up to ~60%. Another example of microfluidic electroporation platforms employs droplet-based physics (Figure 2d) and has shown promise for transfection applications (76, 77). However, due to their design complexity and the need for downstream phase separation, such devices have not been widely implemented. Practically speaking, most microfluidic electroporation platforms have not been implemented at a large scale due to costly fabrication processes, yet they can benefit smaller-scale research efforts, particularly in hard-to-transfect cell types.

Recently, researchers developed a low-cost microfluidic electroporation device (78) that does not require sophisticated fabrication processes such as soft lithography, CNC (computer numerical control) machining, or 3D printing. Given its cost effectiveness, this device could be adapted for mammalian cell applications by redesigning the transfection region and the associated buffers.

#### 3.3.2. Nanostructure-mediated electroporation.

The advent of nanoscale engineering has led to the integration of nanomaterials and nanostructures with microfluidic channels to locally enhance electric fields for single-cell electroporation. Nanostructure-mediated electroporation techniques, unlike conventional cuvette and flow-through microfluidic devices, are intended for more targeted electroporation with highly controlled payload delivery with minimal cell membrane disruption. Boukany et al. (79) reported an early nanochannel-assisted electroporation device in which two microchannels were bridged by an ~90-nm-diameter nanochannel. Applying an electrical pulse across the two microchannels produced a locally amplified electric field within the nanochannel region, and because of the size difference between the cell and the nanochannel, the amplified electric field acted over a relatively small area of the cell membrane. This technique minimally disrupts the cell membrane while facilitating cargo delivery into the cytoplasm, potentially improving cell viability. Boukany et al. achieved controlled cargo delivery by adjusting the electrical parameters, including the number, duration, and magnitude of the pulse.

To improve throughput, investigators have developed devices featuring 2D (80) or 3D (81) arrays of nanochannels (or nanopores) to enable parallel electroporation of multiple cells at a time. Nanostraws, which integrate arrays of hollow, needlelike nanostructures with microfluidic channels (Figure 2g), are another form of nanopore-based electroporation (82, 83). Once an electrical pulse is applied, a locally enhanced electric field is generated across the nanostructures, leading to payload delivery with minimal cell membrane disruption. Upon optimization, nanostraw-assisted electroporation devices can transfect many hard-to-transfect primary cell types, such as human induced pluripotent stem cell–derived cardiomyocytes, human embryonic stem cells, fibroblasts, and mouse primary glial cells, with transfection efficiencies ranging between 60% and 80%.

To achieve more-targeted, spatially controllable payload delivery at the single-cell level, researchers have created a modified nanopore-assisted electroporation platform that integrates with an atomic force microscope (AFM) (84–86), termed nanofountain electroporation (Figure 2h). The probe of the AFM was designed with a submicrometer opening connected to a hollow microchannel intended for payload storage and transport. The probe can easily be brought into contact with any position on the target cell membrane in a spatially controllable manner. Once in contact, an electric field is applied to locally permeabilize the cell membrane, opening pores on the membrane ranging between 1 and 100 nm, depending on the pulse strength. The small pores, coupled with a carefully tuned flow rate, result in a precise payload dosage with minimal damage to the cells (84, 86).

Although these nanomaterial- and nanostructure-mediated techniques can achieve high transfection efficiencies, they are intrinsically batchwise processes, limiting their throughput and scalability. Furthermore, most involve costly, sophisticated, and complex fabrication processes. Moreover, additional equipment may be needed to evenly distribute cells over the nanostructured surfaces, and the payload size is limited by the size of the nanostructures implemented. These issues need to be overcome before these platforms can be widely adopted.

## 4. IN VIVO CELL TRANSFORMATIONS

Reversible and irreversible transformation of cells have become indispensable in medicine, particularly drug delivery and oncology. Some methods for transforming cells include cryosurgery, which uses extreme cold to freeze off abnormal tissues (87); high-intensity focused ultrasound, which uses focused waves to heat and ablate tissues (88); radio-frequency ablation (RFA); and microwave ablation (MWA), which uses heat to burn off a section of a nerve to relieve pain (89). These mechanisms, which utilize thermal effects to kill cells, constitute the current standard of care for several types of cancer. However, the use of extreme temperatures in these techniques has limitations, such as the need to avoid damaging major blood vessels, which could lead to a risk of hemorrhaging and increase the likelihood of metastasis (90). Nonthermal alternatives include iontophoresis, in which a voltage gradient is applied to the skin in conjunction with a charged drug to push molecules across the normal dermal barrier, and electroporation, which can be used both reversibly (to increase cell membrane permeability for DNA or drug delivery) and irreversibly (to ablate unwanted tissues).

In the clinical setting, both RE and IRE techniques typically involve the placement of two to four electrode needles in or around the area of interest to deliver a series of ultrashort (nanoseconds to microseconds) high-voltage pulses. Voltages in the range of 100 to 1,000 V produce reversible effects, whereas voltages up to 3,000 V produce irreversible pores (91). In particular, the extent of electroporation is dependent on electric field distributions and specific electric field thresholds, which are on the order of ~250 V/cm and ~500 V/cm for traditional 100-μs pulses of RE and IRE, respectively (92).

### 4.1. Reversible Techniques

**Figure 3:**
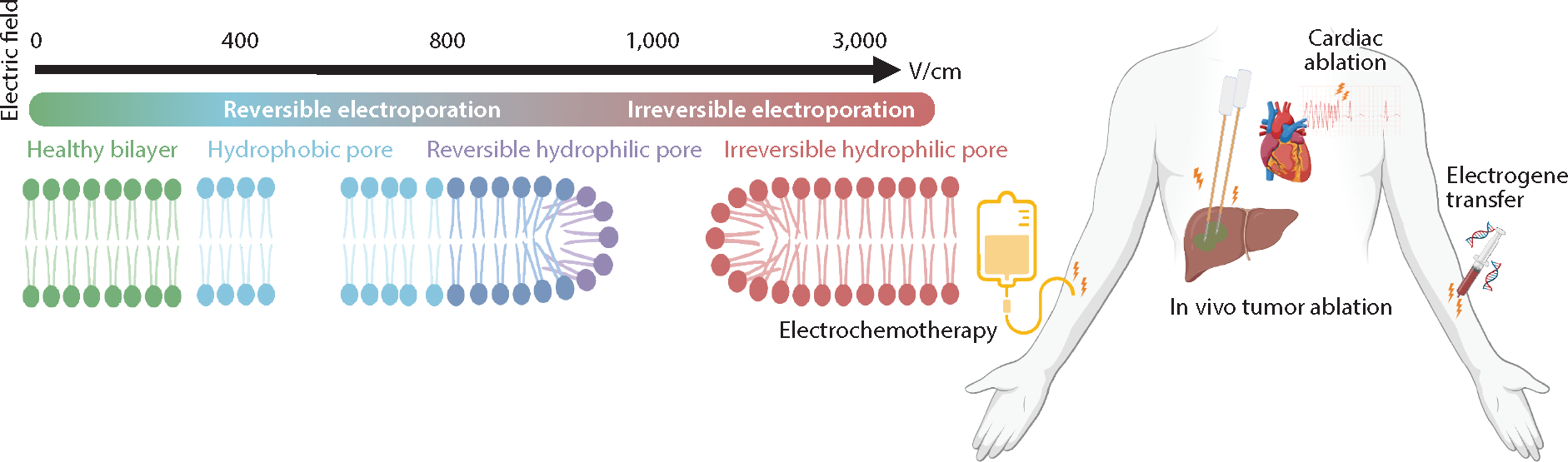
An overview of in vivo electroporation-based techniques. Reversible techniques include electrochemotherapy as an adjuvant to therapeutics and electrogene transfer for gene manipulation to treat a variety of conditions. Irreversible techniques include tissue ablation as a monotherapy for targeting malignant tissues and cardiac ablation for treating arrhythmias. Figure adapted from images created with BioRender.com.

A transient increase in membrane permeability that causes a disrupted cell to regain homeostasis is termed reversible electroporation (RE). A typical pulse sequence for RE consists of eight 100-μs square waves followed by up to eight 100-ms low-voltage square waves for DNA delivery (91). Current clinical modalities of RE-based therapies include ECT and EGT, shown in Figure 3 alongside IRE-based therapies.

#### 4.1.1. Electrochemotherapy.

Electroporation was first clinically utilized in vivo to promote the uptake of chemotherapeutic agents into tumor cells (93); the most-developed ECT drugs are bleomycin (BLM) and cisplatin (CDDP). Combining electroporation with BLM or CDDP improves cytotoxicity 1,000- and 80-fold, respectively (94–96).

In 1987, Okino & Mohri (93) became the first to combine electric pulses with a chemotherapeutic agent. They combined a single 2-ms, 5,000 V/cm pulse following BLM administration in Donryu rats affected with hepatocellular carcinoma (HCC). This concomitant protocol reduced tumor size by 17% from the control. Mir et al. (97) later termed this process electrochemotherapy (ECT) following their experiments in which eight 100-μs, 1,500 V/cm electrical pulses significantly reduced tumor size and even completely eradicated tumors in nude mice. ECT has since become a mainstream local ablative tumor therapy that, as of early 2023, has been used primarily to target skin cancers or skin metastases. Because previous reviews focused on ECT’s primary applications, in the following subsections we focus on its emerging applications.

#### 4.1.2. Emerging electrochemotherapy studies for deep-seated tumors.

While the primary target sites for ECT have been small superficial tumors, ECT has recently shown promise in practice during open surgery procedures. Efforts to expand the therapy to the treatment of deep-seated malignancies percutaneously are underway.

##### 4.1.2.1. Liver cancer.

**Figure 4:**
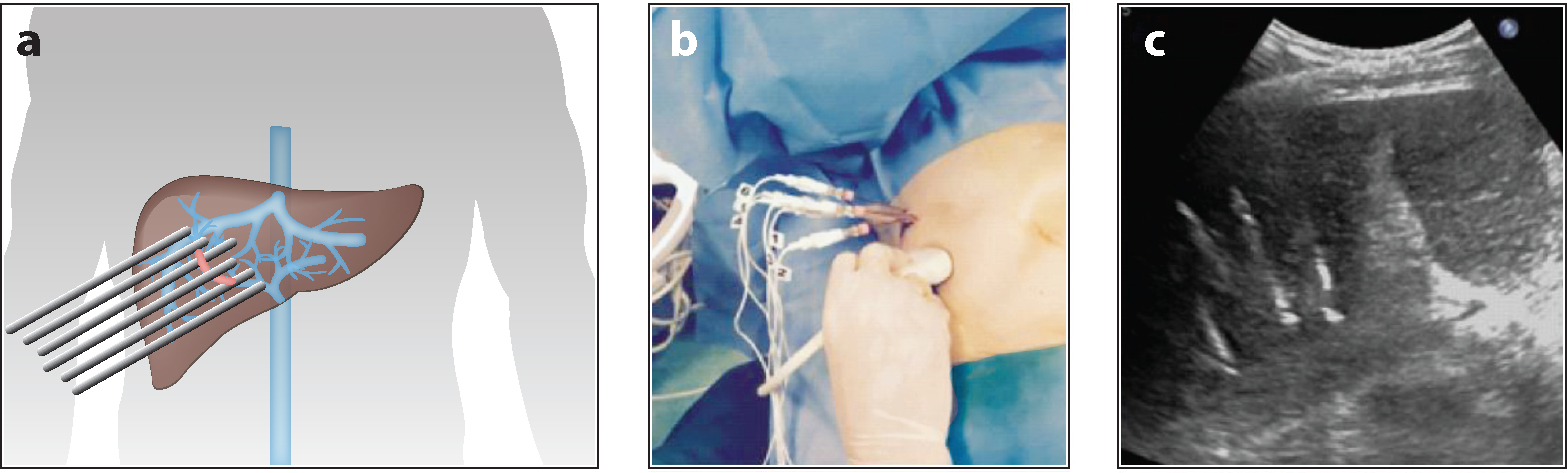
(*a*) Schematic demonstrating the insertion of six electrodes around the margin of tumor thrombosis for electrochemotherapy treatment of the right portal vein. (*b*) Percutaneous insertion of electrode needles via (*c*) ultrasound guidance in a minimally invasive procedure. Figure adapted from Reference 102 (CC BY 4.0).

Liver cancer is the seventh most common cancer worldwide and was the second highest cause of cancer-related deaths in 2020 (98). Liver malignancies are usually targeted through surgical resection along with other treatment options, including cryoablation, RFA, and MWA—all three of which are thermal therapies that pose significant risks around critical arteries and veins. ECT has proven to be safe and effective for treating deep-seated metastases and HCC when conducted in an open procedure (99–101). While open procedures carry more risk and require longer hospitalization, percutaneous ECT approaches have also been investigated. Tarantino et al. (102) reported the first case of image-guided percutaneous ECT on portal vein tumor thrombus (PVTT) from patients with HCC, where electrode needles were positioned by ultrasound guidance (Figure 4). Despite the poor prognosis for HCC patients with PVTT (~3-month median survival), four patients had no local recurrence of the treated PVTT or HCC nodule at the 9–20-month follow-up. Additionally, this study showed complete necrosis in all PVTT lesions, and five of six patients showed necrosis of the HCC tumor nodule. Djokic et al. (103) treated a large, 18-mm^2^ deep-seated HCC under stereotactic cone-beam computed tomography (CBCT) guidance and successfully managed a 36-mm^2^ ablation necrosis area within the treatment zone. Eighteen months posttreatment, the lesion still showed full response. However, both studies had patients in which distant HCC foci appeared.

Despite these distant recurrences, ECT remains advantageous in comparison to alternative options as a nonthermal targeting approach that allows for treatment in proximity to large vessels, such as the hepatic vein and artery, while circumventing adverse heat-sink side effects. A comparative study (104) evaluating treatment of primary and secondary liver malignancies with different alternative treatment options demonstrated 12-month local tumor control percentages as follows: RFA, 93%; ECT, 81%; cryoablation, 70%; interstitial brachytherapy, 68%; and MWA, 61%. Notably, most lesions treated with ECT were significantly larger (volume <20 cm^3^) than those treated with RFA (volume <10 cm^3^). Additionally, 91% of lesions treated with ECT were considered to be located in a challenging region compared with 34% of lesions treated with RFA. Local success in treating lesions in challenging areas in combination with a significant reduction in hospitalization time suggests a positive outlook for percutaneous ECT in treating unresectable deep-seated liver tumors.

##### 4.1.2.2. Pancreatic cancer.

Most pancreatic cancers are derived from cells that line the duct of the pancreas, and they are often classified as pancreatic adenocarcinomas (105). Adenocarcinoma is an aggressive cancer often treated through surgical resection and chemotherapy, when possible. However, approximately 40% of local tumor structures are encased within critical vascular structures, eliminating the possibility of safe resection (106).

The dense stromal layer of the tumor often diminishes the efficacy of administered chemotherapeutics. However, because ECT is capable of disrupting this impenetrable layer, it is a favorable option for overcoming this barrier and enhancing therapeutic outcomes. One study (107) demonstrated the safety and feasibility of ECT on locally advanced pancreatic cancer (LAPC) and reported no major side effects or complications. The 25 patients in this study who received ECT during an open procedure showed no evidence of intraoperative bleeding or damage to surrounding internal organs, with the exception of three patients with venous stasis of the duodenum. Interestingly, all of these patients had biliary stents. Metal stents in proximity to large electric fields can conduct or deflect large amounts of energy, leading to incomplete ablations and even thermal injury (108).

ECT can improve or relieve the pain that often accompanies LAPC and may be considered a feasible palliative treatment for these otherwise unresectable tumors. Percutaneous ECT approaches for the pancreas have been conducted only in preclinical animal trials through CBCT guidance (109); however, a percutaneous approach for IRE utilizing a similar electrode setup has been tested in humans and determined to be safe and feasible (110). Despite this success, another study with the same setup for IRE under ultrasound guidance showed a high complication rate (111), demonstrating the need for further investigation of percutaneous approaches.

##### 4.1.2.3. Brain cancer and other intracranial diseases.

The brain presents a unique challenge for delivering chemotherapeutic agents to treat brain tumors. The blood–brain barrier (BBB) is a highly selective barrier composed of tight junction proteins that regulate the transport of molecules and pathogens between the circulatory and central nervous systems. While critical for keeping infection-causing toxins from entering the brain, the BBB also makes it difficult for large therapeutic molecules to pass through. However, electrical pulses can focally and transiently permeate the endothelial lining of the BBB, enabling passage of various drug molecules (112). Notably, the mechanism of BBB disruption is physiologically different from that of RE. BBB disruption involves the disruption of tight junction proteins (113), while RE is based on exceeding a set TMP of the cellular membrane. However, the ability to induce RE in tumor tissue while drugs bypass the BBB shows promise for targeting enhanced drug uptake in tumor infiltrates beyond the target tumor ablation zone. In 1993, Salford et al. (114) became the first to investigate ECT for primary brain tumors. Tumor-bearing rats treated with a two-needle configuration and BLM had a survival rate double that of rats receiving BLM alone. Later, Agerholm-Larsen et al. (115) used a new electrode configuration, with electrodes expanding outward after insertion, to expand treatment zones through a single burr hole. The authors inserted either four or eight electrodes into tumor-bearing rats. The eight-needle electrode configuration produced better morphologic changes in brain tissue, including necrosis, macrophage invasion, and tumor elimination, likely attributable to the expansive usable electric field. Treatment was localized, and 69% of the treated tumors resolved. Different electrode configurations and pulsing waveforms may be used to fine-tune low-field (BBB disruption) distributions while minimizing high-field (ablation) exposure (116).

ECT applications in the brain warrant further research. Researchers may consider using preclinical canine models for these brain studies; canines commonly produce spontaneous gliomas that display many of the same important clinical and pathological features as those of humans, making them a valid translational model for treatment.

#### 4.1.3. Electrogene transfer.

Genetic material can be transferred into a cell under the influence of electroporation. In this process, termed electrogene transfer (EGT), gene electrotransfer, or DNA electroporation, plasmid DNA is injected into a target tissue following electric pulses. Current gene delivery systems are based on either viral vectors or nonviral vectors. While viral vectors have a greater transfection efficiency, they have limitations in reproducibility and size of the DNA which can be introduced into a cell. Nonviral systems can overcome these limitations, but their transfection efficiency is limited. Transfection efficiency is challenged by the negatively charged cell membrane, which deflects negatively charged DNA, the dense cytoskeleton network, and the nuclear envelope.

Electroporation-mediated DNA vaccination can help overcome these limitations. The measured uptake of exogenous substances under electric fields is on the order of a magnitude greater than that of methods where electric fields are absent. It is becoming more widely believed that this distinction is due to a two-step process in which (*a*) the initial pulses of electroporation permeabilize the cell and (*b*) pulses encourage electrophoretic migration of DNA through the transient pores of the cell membrane.

Transport of DNA into cells is a useful means of altering the properties of defective cells. While this technique is employed primarily in laboratory transfection assays, its use in clinical settings is still being investigated. As of early 2023, 25 such clinical trials are active or recruiting; they include trials on human papilloma virus–associated cancers, HIV-positive anal lesions, breast cancers, pancreatic cancers, prostate cancers, blood cancers, skin cancers, lung cancers, hepatitis B and C infections, dengue virus, and severe acute respiratory syndrome coronavirus 2 (SARS-CoV-2).

##### 4.1.3.1. Electrogene transfer in oncology.

DNA transfer may boost antitumor properties through the introduction of genes that produce immunogenic proteins. In particular, cytokine gene therapy has recently emerged as a promising therapy for stimulating host immunity against tumor antigens. This treatment has the potential to halt or completely eradicate tumor cell growth. A 1984 study (117) found that interleukin (IL)-2 stimulated the growth of lymphokine-activated killer cells, cytotoxic T lymphocytes, tumor-infiltrating lymphocytes, and natural killer (NK) cells. However, high concentrations of circulating IL-2 are intrinsically toxic. This limitation was overcome by the introduction of IL-2 via plasmid DNA injection mediated by electroporation.

The first in vivo EGT trial evaluated the toxicity and efficacy of IL-12 in metastatic melanoma patients (118). It achieved local regression rates similar to those of other gene delivery approaches, but with greater efficacy against tumor recurrence, suggesting an antitumor immune response.

##### 4.1.3.2. Electrogene transfer for COVID-19 vaccination.

During the SARS-CoV-2 pandemic, researchers investigated the use of EGT to more efficiently deliver COVID-19 vaccines (119). One study showed that while EGT does not improve efficacy in delivering a nonreplicating RNA vaccine (120), EGT could significantly improve the outcomes of DNA vaccines and replicating RNA vaccines (121). Electroporation-mediated vaccination significantly increased uptake of the COVID-19 DNA vaccine Inovio INO-4800 and promoted the formation of T cells protecting against invaders and B cells working to neutralize the virus. A lack of readily available electroporating equipment and the need to train medical staff for mass vaccination have made EGT adoption less favorable for emergency use.

### 4.2. Mechanisms of Cell Death

Electroporation-based treatments induce different types of cell death on the basis of pulsing parameters, tissue types, and adjuvant-mediated electroporation. Given that RE alone does not induce cell death, variability in mechanisms is attributed to chemotherapeutics, organ site, and dosing (122). Stimulation of the sympathetic nervous system during ECT causes vascular lock, leading to immediate vasoconstriction of the blood supply to the stimulated area. This effect lasts longer in neoplastic areas, enhancing tumor exposure to drugs and in turn making chemotherapeutic-induced mitotic cell death the most potent mechanism in ECT (123). Apoptosis and pyroptosis have also been commonly observed in BLM-mediated ECT, and necroptosis has been observed in both BLM and CDDP ECT (124, 125).

Nanosized tears in the cell membrane also trigger cell death. Early IRE studies suggested that apoptosis was the primary mode of cell death, but many of these studies did not consider alternative mechanisms. Necrosis occurs in high–electric field regions (>3,000 V/cm) (126, 127), necroptosis in moderately high fields (1,000–3,000 V/cm), and apoptosis in fields approaching the outer regions where RE occurs. High-frequency bipolar bursts have shown greater immunogenic responses, such as necroptosis and pyroptosis (128, 129).

### 4.3. New Frontiers in Electroporation

As electroporation-based therapies including ECT, EGT, and IRE continue to evolve in the clinic, newer applications have been expanding into both new and established treatment avenues in cancer care. In the following subsections, we discuss the integration of electroporation with modern cancer immunotherapies and established radiotherapy protocols, as well as an emerging application in calcium electroporation (CaEP).

#### 4.3.1. Electroimmunotherapy.

Immunotherapy has recently commanded the attention of researchers and clinicians across various fields. While electroporation opens the cell membrane to allow drug molecules in, intracellular proteins are also able to escape, acting as damage-associated molecular patterns that can alert the body to vulnerability once they escape the cell and are exposed to the extracellular environment (130). A critical advantage of electroporation-based immune response induction over thermal-based techniques is the ability to release these antigens without denaturation. This enhancement in the immunogenic response results in an elevated CD8^+^ T cell response that eradicates primary tumors, untreated distant tumors, and metastases. Immunodeficient mice treated with ECT had significantly less tumor regression than immunocompetent mice (131), corroborating the idea that the immune system is crucial to the efficacy of ECT. Following ECT, B cells, NK cells, NK T cells, macrophages, and dendritic cells infiltrate the intratumoral space (132), while the number of Tregs decreases.

Through the use of EGT, researchers have been able to overcome the limitation of adverse reactions from high-dose cytokine therapies. Control over kinetics and expression levels enables the delivery of concentrations of plasmid DNA through EGT while maintaining its efficacy and prolonging the presence of the therapeutic agent. Additionally, studies have investigated the combined use of ECT and EGT; ECT targets the local tumor, while cytokine delivery by EGT increases the systemic effects of ECT in order to eradicate nonprimary lesions and prevent the formation of new tumors and metastasis via immune system activation (133, 134). Due to the nonthermal nature of electroporation-based therapies, the ability to preserve functional blood vessels and greater facilitation of CD8^+^ T cell infiltration may make the electroporation immune response more prominent than in thermal- or radiation-based therapies. Even when thermal damage is unlikely, thermal mitigation techniques may be incorporated to reduce even mild to moderate thermal effects in order to better promote immune activation, in turn improving patient outcomes (122, 135, 136). With all forms of electroporation-based therapies showing a positive influence on immune stimulation, further studies investigating combinations of different electroporation-related therapies should be pursued.

#### 4.3.2. Electroporation as a radiosensitizer.

Radiotherapy is the standard of care for many malignant tumors. However, because of its inability to discriminate between malignant and healthy phenotypes, researchers have explored methods to increase the radiosensitivity of the malignant phenotypes. These methods include radiosensitizing drugs such as BLM and CDDP. The combination of ECT and irradiation increases the radiosensitizing effect of BLM and CDDP by 1.9-fold and 1.26-fold, respectively, in in vivo studies (137, 138). Additionally, even electroporation alone was observed to lead to increased radiosensitivity. The mechanism by which this process occurs is not entirely understood; however, a possible explanation is that the increase in intracellular aqueous fluids, increased free radical damage, inhibition of single-strand-break repair, and delayed DNA repair all enhance the failure to repair DNA (139). One study found that a single 100-μs pulse delivered 10 min before irradiation increased radiosensitivity by a factor of 1.18; the window of radiosensitivity was significant for up to 50 min, allowing for a realistic time frame for patients to receive a safe dose of electroporation followed by irradiation in a clinical setting (140). Additionally, electroporation may be used as a radioenhancer, enabling a reduction in the radiation dose required to kill malignant cells. A single-site feasibility trial for patients with intermediate-risk prostate cancer, in which patients will undergo IRE followed by magnetic resonance–guided radiotherapy, is currently recruiting (see https://clinicaltrials.gov/ct2/show/NCT05345444). It is expected that the combination of IRE with radiation therapy will prove to be safe and feasible, providing optimal treatment for these patients.

#### 4.3.3. Calcium electroporation.

CaEP is a new method in which high concentrations of calcium are delivered into the cell through electroporation. Calcium in the mitochondria acts as an intracellular second messenger involved in cell processes including proliferation, differentiation, and cell death (141, 142). While calcium flows freely in the outer membrane, it requires assistance from ion channels and transporters to travel through the inner membrane.

Similar to ECT, CaEP involves injection of calcium into the intratumoral space followed by electroporation, causing calcium to pass from the extracellular to the intracellular space. The high influx of calcium leads to a mitochondrial permeability transition. This transition disrupts the permeability of the inner mitochondrial membrane, resulting in a loss of ATP production from the disrupted mitochondria and a higher consumption of ATP in an attempt to restore homeostasis. This loss of ion homeostasis and ATP depletion result in cell death by necrosis or apoptosis. Cell death may also be due to the formation of reactive oxygen species, lipases, and proteases (143).

CaEP can be used to terminate expression of a transgene injected during EGT in the case of an adverse event (144). In 2012, investigators first proposed the use of CaEP as an anticancer technique (145). Since then, many studies, including a clinical trial, have found that CaEP is comparable to ECT for cutaneous tumors (146).

## 5. CONCLUSION

Interest in nonviral transfection methods is on the rise, with electroporation being used for both basic research and clinical applications. For tumor control, cell permeabilization has demonstrated success in facilitating localized chemotherapeutic uptake while minimizing systemic toxicity. The use of percutaneous noninvasive electroporation techniques may help produce shorter procedure times, shorten inpatient hospital stays, and promote utilization of fewer electrodes, resulting in lower cost and greater patient convenience.

## Abbreviations

Necrosis: moderately proinflammatory accidental and rapid cell death
Apoptosis: noninflammatory programmed cell death
Pyroptosis: highly proinflammatory programmed cell death
Necroptosis: moderately proinflammatory slow programmed cell death
Cancer immunotherapy: making use of one’s own immune system to detect and fight off malignant cells that would otherwise be undetected due to the immunosuppressive tumor environment
